# Dendritic cell vaccination combined with carboplatin/paclitaxel for metastatic endometrial cancer patients: results of a phase I/II trial

**DOI:** 10.3389/fimmu.2024.1368103

**Published:** 2024-02-20

**Authors:** Bouke J. Koeneman, Gerty Schreibelt, Mark A. J. Gorris, Simone Hins - de Bree, Harm Westdorp, Petronella B. Ottevanger, I. Jolanda M. de Vries

**Affiliations:** ^1^ Department of Medical BioSciences, Radboudumc, Nijmegen, Netherlands; ^2^ Department of Medical Oncology, Radboudumc, Nijmegen, Netherlands

**Keywords:** endometrial cancer (EC), immunotherapy, chemoimmunotherapy, DC vaccination, dendritic cells

## Abstract

**Background:**

Metastatic endometrial cancer (mEC) continues to have a poor prognosis despite the introduction of several novel therapies including immune checkpoints inhibitors. Dendritic cell (DC) vaccination is known to be a safe immunotherapeutic modality that can induce immunological and clinical responses in patients with solid tumors. Platinum-based chemotherapy is known to act synergistically with immunotherapy by selectively depleting suppressive immune cells. Therefore, we investigated the immunological efficacy of combined chemoimmunotherapy with an autologous DC vaccine and carboplatin/paclitaxel chemotherapy.

**Study design:**

This is a prospective, exploratory, single-arm phase I/II study (NCT04212377) in 7 patients with mEC. The DC vaccine consisted of blood-derived conventional and plasmacytoid dendritic cells, loaded with known mEC antigens Mucin-1 and Survivin. Chemotherapy consisted of carboplatin/paclitaxel, given weekly for 6 cycles and three-weekly for 3 cycles. The primary endpoint was immunological vaccine efficacy; secondary endpoints were safety and feasibility.

**Results:**

Production of DC vaccines was successful in five out of seven patients. These five patients started study treatment and all were able to complete the entire treatment schedule. Antigen-specific responses could be demonstrated in two of the five patients who were treated. All patients had at least one adverse event grade 3 or higher. Treatment-related adverse events grade ≥3 were related to chemotherapy rather than DC vaccination; neutropenia was most common. Suppressive myeloid cells were selectively depleted in peripheral blood after chemotherapy.

**Conclusion:**

DC vaccination can be safely combined with carboplatin/paclitaxel in patients with metastatic endometrial cancer and induces antigen-specific responses in a minority of patients. Longitudinal immunological phenotyping is suggestive of a synergistic effect of the combination.

## Introduction

Endometrial carcinoma (EC) is the most common gynecological malignancy in the western world, and currently the only one with both a rising incidence and mortality (1). The majority of patients are diagnosed with stage I disease, which is associated with a 5-year survival rate of over 90% (2). Treatment for early-stage disease consists of surgery with or without adjuvant radiotherapy or chemotherapy, dependent on risk-stratification based on clinicopathological and molecular features (3). In contrast with the favorable prognosis for most patients with early-stage disease, the 5-year survival rate for metastatic EC (mEC) at diagnosis remains below 20%, reflecting a lack of major advances in systemic therapies for this disease (4).

A subgroup of mEC patients with mismatch repair deficient (dMMR)/microsatellite instable (MSI) or POLE ultra mutated tumors responds well to immune checkpoint inhibition (ICI). Clinical results with ICI monotherapy in other mEC patients (70-80% of cases) are less favorable (5–8). The association between the presence of tumor infiltrating lymphocytes and a favorable prognosis (9) suggests that immune-based therapy holds promise for improved care for these patients for whom checkpoint inhibition (alone) might not be the ideal modality.

Therapeutic dendritic cell (DC) vaccination is an immunotherapeutic approach to immunotherapy that could be a rational adjunct in the treatment of these patients. DCs are the most potent antigen presenting cells of the immune system and form the bridge between the innate and adaptive immune system (10). In DC vaccination, autologous DCs are loaded with relevant tumor antigens *ex vivo* and administered to the patient with the aim of inducing specific T-and B-cell responses. Over 200 clinical trials show that its use is safe and remarkable clinical responses are observed (11). To improve the response rate for this type of immunotherapy it is crucial to optimize the vaccine composition as well as the mode of administration and timing in relation to other therapies.

The first vaccine composition-related factor to consider is the source of autologous DCs. Until recently, mostly monocyte-derived DC (moDC) have been used in clinical trials. A disadvantage of this approach is that the conversion of blood monocytes into moDC requires an extensive culture period and the use of several compounds that diminish cell functionality. Nowadays, it is possible to purify fully functional natural DCs (nDCs) directly from blood using Good Manufacturing Practices (GMP)-compliant immunomagnetic isolation (12). Several DC subsets exist, the main distinction being between plasmacytoid (pDC) and conventional DCs (cDCs, sometimes called myeloid DCs). Conventional DCs can be further subdivided based on expression of surface markers in the rare conventional DC type 1 (cDC1), the most abundant type 2 (cDC2), and the recently described type 3 (cDC3), each with its own function (12, 13). In early nDC vaccination studies either cDC2 alone (14) or the pDC alone (15) were used. Later studies with the combination of conventional and plasmacytoid DCs observed a synergistic effect of these two subsets (16–18). The cDC1 subset is of particular interest for immunotherapy due to its capability for cross-presentation, but its use in clinical trials so far has been hampered by the small numbers in which it is present in peripheral blood (19). This is the reason cDC2s were used in this and in previous studies. A second essential factor for vaccine efficacy is choosing appropriate antigens for DC loading. Most DC vaccination trials have used either purified tumor-associated protein or whole tumor lysate for antigen loading. None of these sources has yet proven to be superior to the other as both have their advantages and disadvantages (20). In this study we loaded cDC2s and pDCs with PepTivators covering the complete proteins Mucin-1 and Survivin, known immunogenic EC targets (21–24).

The importance of the route of administration of a DC vaccine was demonstrated in migration studies that showed that, upon intravenous or intradermal injection, most cells do not reach the lymph node, the site of T-cell activation (25). We have established ultrasound-guided intranodal injection as a reliable method to deliver DCs directly to their site of action (26).

Optimal integration of DC vaccination within treatment schedules is the final critical factor for success. Vaccination efficacy can be hampered by immune evasion strategies employed by malignant cells, some of which can be overcome by combining therapies. The presence of myeloid derived suppressor cells (MDSC) and regulatory T-cells (Tregs) in the tumors protect malignant cells from immune eradication (27). We and others have recently shown that platinum-based chemotherapy strongly reduces MDSC counts as well as their suppressive function (28, 29). Cisplatin reverses STAT6-mediated upregulation of PD-L2 *in vitro (* 30), although the clinical benefit of this combination has not yet been confirmed (31). These observations of possible synergistic benefit provide the rationale for combining nDC vaccination with chemotherapy.

This study aims to show that the combination of a peptide-loaded nDC vaccine with carboplatin-paclitaxel chemotherapy is safe and feasible and can induce an anti-tumor immune response in endometrial cancer patients that ultimately could lead to better disease control without additional toxicity.

## Materials and methods

### Study design

We conducted the DECENDO study, a single-arm phase I/II study to show immunological efficacy of nDC-vaccination in mEC patients receiving palliative chemotherapy. The trial protocol was approved by the Dutch Central Committee on Research involving Human Subjects and registered at clinicaltrials.gov (NCT04212377). The study population consisted of 7 patients ≥18 years of age with stage IV EC who could not receive hormonal therapy and who were eligible for treatment with carboplatin/paclitaxel chemotherapy. Written informed consent was obtained from each patient. Other important inclusion criteria were WHO/ECOG performance status 0-1, adequate bone marrow, liver, and kidney function as demonstrated by laboratory testing and exclusion of pregnancy for participants of childbearing potential. All patients were required to express Survivin and/or Mucin-1 on their tumor material, as these antigens were used to load DCs during vaccine preparation. We excluded patients who had a history of any other malignancy in the previous 5 years, heart failure (NYHA class III/IV) serious active infections, autoimmune diseases, or used systemic corticosteroids. Clinical endpoints were safety and feasibility of nDC vaccinations. Safety was assessed using Common Terminology Criteria for Adverse Events (CTCAE) version 4.0. Response evaluation was done according to the Response Evaluation Criteria in Solid Tumors (RECIST) version 1.1.

### Treatment schedule

**Figure 1:**
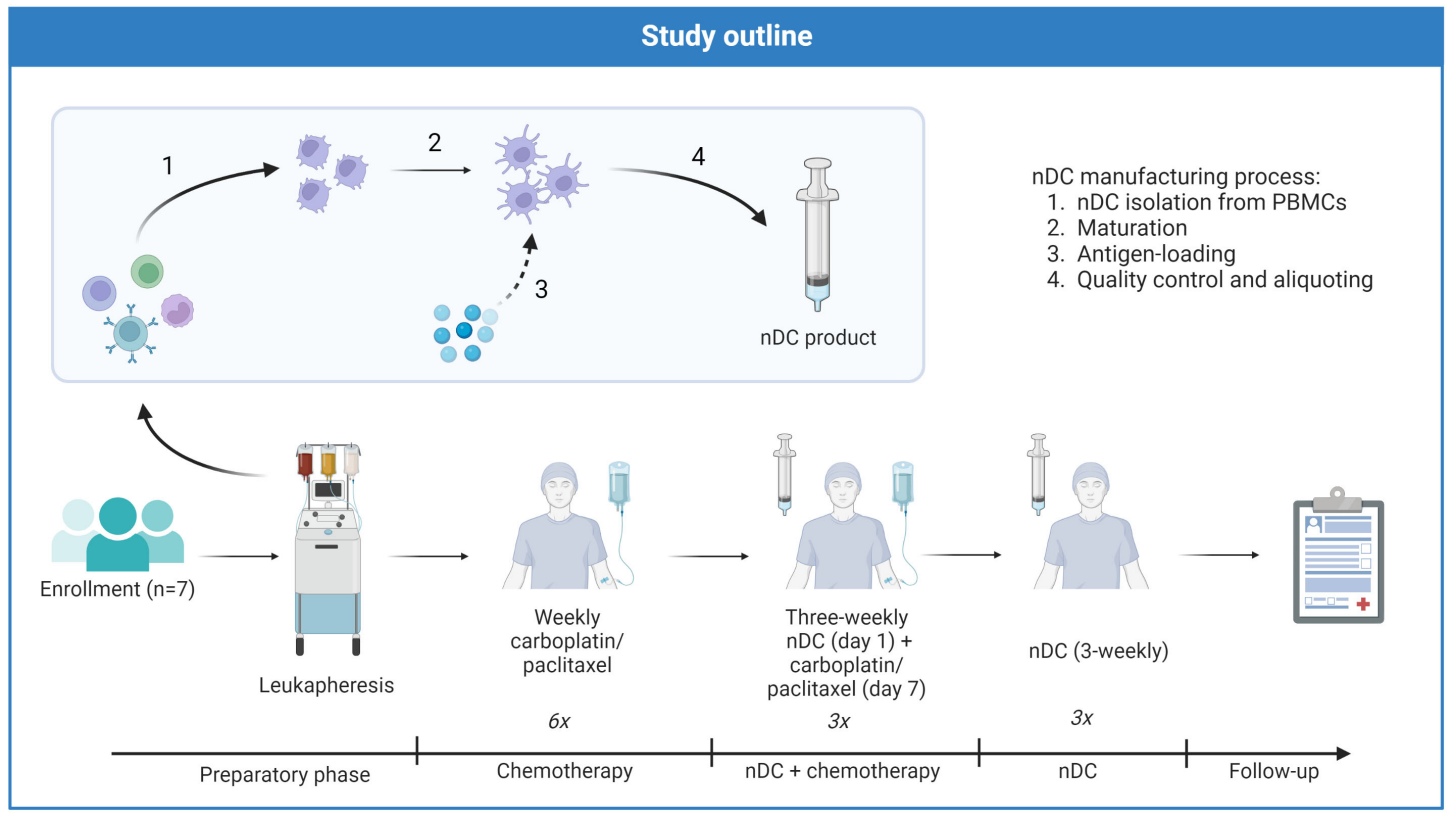
Treatment schedule of DECENDO. nDC natural dendritic cells, PBMCs peripheral blood mononuclear cells.

An overview of the treatment schedule is provided in **Figure 1**. nDC vaccinations were incorporated in a chemotherapy regimen previously described by Van der Burg et al, with weekly followed by three-weekly carboplatin/paclitaxel (32). After a preparatory phase, during which leukapheresis was performed and nDC manufacturing commenced, patients went through three subsequent treatment phases: chemotherapy alone, nDC vaccinations + chemotherapy, and finally nDC vaccinations alone. The first two phases were followed by disease evaluations, patients only advanced to the next phase if they did not have progressive disease (PD). The first phase of initial chemotherapy consisted of six weekly administrations of carboplatin (AUC 4) and paclitaxel (90 mg/m^2)^). The second phase started one week after the last day of chemotherapy in the previous phase and involved combined chemoimmunotherapy: three three-weekly cycles with DC vaccination on day one and carboplatin (AUC 5) and paclitaxel (175 mg/m^2^) on day seven. The third and last phase comprised three additional nDC vaccinations without chemotherapy, continuing the pattern of one vaccination every three weeks. Subsequently, patients were closely monitored during 12 months follow-up and afterwards throughout the regular follow-up with CA-125 measurements and CT scans according to standard of care.

### Vaccine preparation and administration

nDCs were purified from the apheresis product with the fully automated and closed immunomagnetic CliniMACS Prodigy^®^ isolation system (Miltenyi Biotec) using a combination of positive and negative selection with magnetic bead-coupled antibodies (Miltenyi Biotec). Negative selection consisted of depleting monocytes and B-cells using magnetic bead-coupled CD14 and CD19 antibodies, respectively. Positive selection for pDCs was done using magnetic bead-coupled anti-BDCA4. cDC2s were first labeled with anti-BDCA1-biotin and subsequently selected with magnetic bead-coupled anti-biotin antibodies. The resulting purified nDCs were cultured overnight in 6 wells plates at 37°C 5% CO_2_ at a concentration of 1.5 x 10^6^ cells/ml with 800 IU/ml recombinant human GM-CSF and 10ng/ml recombinant human IL-3 in TexMACS GMP medium (all Miltenyi Biotec) supplemented with 2% human serum (Sanquin, Amsterdam, the Netherlands), 10 µg/ml keyhole limpet hemocyanin (KLH) (Immucothel, Biosyn Arzneimittel GmbH, Fellbach, Germany) for immunomonitoring and MACS^®^ GMP-grade PepTivators^®^, overlapping peptide pools of Survivin and Mucin-1 (PepTivator^®^, Miltenyi Biotec).

After overnight culture, a maturation step followed during which nDCs were activated for 6h with 15 μl/ml premixed protamine/mRNA (Protamine HCl, 5000 IU/ml = 50 mg/ml, Meda Pharma, Amstelveen, the Netherlands, mixed with 5 μg mRNA, Universitätsklinikum Erlangen, Erlangen, Germany for 10 minutes). 3 hours into this maturation step, viability and phenotype were assessed. After 6h of maturation, cells were washed twice with NaCl 0.9% supplemented with 5% Albuman (final concentration) and cryopreserved in TexMACS medium containing 10% dimethyl sulfoxide (DMSO; WAK Chemie Medical GmbH, Steinbach, Germany) and 40% Albuman (Sanquin). Vaccines were stored below -150°C, and thawed on the day of administration. To ensure sterility, viability, purity, and proper maturation status of the nDC product, quality control was performed. nDCs were required to meet the following release criteria: sterile (tested by Eurofins Bactimm, Nijmegen, the Netherlands), free of endotoxins, ≥ 80% viability, ≥ 30% CD80 expression, ≥ 50% CD83 expression, ≥ 50% CD86 expression, ≥ 90% MHC class I expression, ≥ 90% MHC class II expression, ≥ 30% CCR7 expression, and a potency index >2.0. The potency index was defined as T cell activation in a mixed lymphocyte reaction of peripheral blood lymphocytes (PBLs) with mature nDCs/T cell activation of control PBLs without nDCs. The nDC product was administered (3-8x10^6^ cells per dose) in an inguinal lymph node under ultrasound guidance by an experienced radiologist.

### Collection of biomaterials

Peripheral blood for phenotyping of circulating immune cells was collected before leukapheresis and on the day of the third and sixth vaccinations. PBMCs were isolated using Ficoll-separation prior to storing in liquid nitrogen until analysis.

Delayed type hypersensitivity (DTH)-skin testing was used to test for presence and functionality of antigen-specific T-cells, using a protocol described in more detail elsewhere (33). Briefly, activated peptide-loaded nDC (5 x 10^5^ nDC) were injected intradermally. Two days later, a skin biopsy was taken from which skin-test infiltrating lymphocytes (SKILs) were cultured.

An image-guided biopsy of a metastatic lesion was performed and formalin-fixed and paraffin-embedded (FFPE) for immunohistochemical (IHC) staining, prior to start of study treatment. Tissue from primary tumors was obtained from archival FFPE resection material.

### Responses to KLH

Cellular responses to the control antigen KLH were analyzed using a proliferation assay. In this assay, PBMCs were cultured in a 96-well plate in either absence or presence of KLH. After four days of culture, cells were incubated for 8-18 hours with tritium-thymidine. Radioactive thymidine uptake as a measure for proliferation was quantified using a microbeta counter. A proliferation index (proliferation of stimulated PBMCs divided by baseline proliferation) of >2 was considered positive.

### Tumor antigen-specific responses

The occurrence of spontaneous or vaccine-induced tumor-specific T-cells against survivin or mucin-1 peptides was assessed in both PBMCs and SKILs obtained from the DTH-skin test using methods described previously. First, in HLA*A02:01 positive patients, cells were stained with fluorescently labeled dextrameric complexes of MHC-I with relevant tumor peptides (Immudex, Virum, Denmark) (33). Two different fluorophores were used for staining and only the double-positive cells of the live CD8 T-cell population were considered antigen-specific to account for non-specific binding. Dextramers with peptides of survivin-1:5-14 (TLPPAWQPFL), survivin-1:95-105 (LTLGEFLKL) and Mucin (LLLLTVLTV) were used. Second, in all patients, functionality of SKILs was tested using a coculture assay with autologous PBMCs that were either pulsed with relevant peptide mixes (Survivin 1 PepTivator, Mucin-1 PepTivator) or unpulsed (16). In brief, cytokine production (IFN-gamma, IL-2, GM-CSF) by T-cells in response to coculture with PBMCs was measured using a cytometric bead array (MACSPlex Cytokine Kit, Miltenyi Biotec) and compared between pulsed and unpulsed PBMCs as a negative control. Based on the sensitivity of the assay and results from previous studies (33), a cytokine concentration of > 50 pg/ml compared to unpulsed PBMCs was considered biologically meaningful.

### Flow cytometry

Flow cytometry was used to determine purity and phenotype of nDCs after immunomagnetic isolation and maturation and to measure the proportion of and expression of activation markers on relevant circulating immune cell subsets in peripheral blood longitudinally. For the peripheral blood samples, three independent multicolor panels were used to quantify DC-, MDSC-, and T-cell subsets. A fourth panel was employed to assess expression of targetable immune checkpoints in circulating T-cells. A full list of the monoclonal antibodies (mAbs) that were used in the different panels is available in **Supplementary Table S1** . Fixable viability dye efluor 780 (eBioscience, San Diego, CA, USA) was used in all panels to exclude dead cells. Where necessary, appropriate isotype or fluorescence minus one controls were used. Stained samples were measured with a FACSVerse^®^, FACSLyric^®^ (BD biosciences, San Jose, CA, USA) or MACS Quant^®^ (Miltenyi Biotec) and data analysis was performed with the Cytobank platform.

### Chromogenic immunohistochemistry for Survivin and Mucin-1

Antigen expression of Survivin and Mucin-1 was assessed on archived FFPE blocks of tumor biopsies taken from metastatic lesions. Mucin-1 staining was performed according to a protocol described previously (17). For Survivin, the procedure was similar. First, 4-μm thickness sections were cut, and slides were deparaffinized and rehydrated prior to antigen retrieval by boiling in EnVision™ FLEX target retrieval solution (pH 9, K8004, Dako) for 10 minutes. Subsequently, endogenous peroxidase was blocked using 3% hydrogen peroxidase (76,051,800.1000, EMD Millipore) in PBS (4391.9010, Klinipath) for 10 minutes. Incubation was performed with a primary anti-Survivin antibody (D8, sc-17779, Santa Cruz Biotechnology, TX, USA, dilution) for 1 hour at room temperature. EnVision™ FLEX Wash Buffer (DM831, Dako) was used to wash between steps. Afterwards, slides were incubated with the secondary antibody BrightVision poly-HRP-anti-Ms/Rb/Rt IgG (DPVO999HRP, ImmunoLogic) at room temperature for 30 min. Finally, incubation with EnVision™ FLEX DAB Buffered Substrate and EnVision™ FLEX Substrate Buffer (K5207 and SM803; DAKO) was done for 10 min at room temperature prior to dehydration, counterstaining with hematoxylin and finally enclosing with Quick-D mounting medium (7281, Klinipath). Finally, a pathologist evaluated antigen expression as either positive or negative.

### Multiplex immunohistochemistry

We used the multiplex immunohistochemistry workflow described in more detail elsewhere (34, 35), to characterize changes in the immune cell infiltrates of tumors over time. In brief, sections were taken from FFPE samples and mounted onto slides. After deparaffinization, epitope retrieval, slides were stained using Opal multiplex IHC Detection Kits. Staining with a multiplex panel was performed with a Bond RX autostainer (Leica Biosystems) using DAPI for nuclear staining as well as primary antibodies directed against CD56, CD8, CD20, CD3, Foxp3 and anti-pan-cytokeratin (details provided in **Supplementary Table S2**). For each target, a primary antibody incubation was followed by incubation with a secondary antibody and finally with an Opal fluorophore.

Image acquisition was performed with the Vectra^®^ Polaris™(PerkinElmer).

InForm^®^ software (Akoya Biosciences) was used for segmentation of tumor and stroma regions in the acquired images. Subsequently, the neural network ImmuNet (36) was employed to detect immune cells and quantify marker expression on each cell. The output of this analysis was converted to Flow Cytometry Standard (FCS) files and cells were phenotyped using FlowJo software (V10, BD Biosciences).

### Statistics

The open-source software Python (Spyder IDE, v 5.3.3) was used for statistical analyses. Descriptive statistics of the immunological response and patient survival data include means, standard deviations, and medians. To compare changes between different timepoints, if applicable, paired t-tests were used if data was normally distributed and Wilcoxon signed-rank tests were used if data was not normally distributed.

## Results

### Patient characteristics

Eight EC patients were enrolled in this exploratory study. All patients expressed both Survivin and Mucin-1 on the available tumor material (representative images of antigen expression are provided in **Supplementary Figure S1**). Seven patients passed screening and underwent apheresis, whereas one patient failed study screening due to rapidly progressive disease. Demographic and tumor characteristics of the seven patients who underwent apheresis are summarized in **Table 1**. Median age was 65 years, all patients already underwent hysterectomy for lower stage EC. Two patients were treated with adjuvant carboplatin and paclitaxel as adjuvant therapy and one for metastatic disease, three patients received prior radiotherapy.

**Table 1:** Baseline clinical and tumor characteristics.

Patient	Age	Prior treatment	WHO performance status	Metastasis location	Mutations in gene(s)	Amplification(s) in gene(s)	Microsatellite instability	PD-L1 status
**Endo-02**	69	Hysterectomy, chemotherapy	**1**	Pulmonary, peritoneal depositions	ERBB2, TP53	FGFR1	No	Negative (0%)
**Endo-03**	68	Hysterectomy, chemotherapy	**0**	Pulmonary, retroperitoneal lymph nodes	KRAS and PIK3CA (activating)	No	No	Unknown
**Endo-04**	60	Hysterectomy, radiotherapy	**1**	Mesenteric lymph node	**-**	**-**	**-**	**-**
**Endo-05**	65	Hysterectomy, chemotherapy	**1**	Pulmonary, thoracic wall	PTEN, TP53	No	Yes	Unknown
**Endo-06**	71	Hysterectomy	**0**	Mediastinal lymph nodes, pelvic cavity	–	–	–	–
**Endo-07**	58	Hysterectomy, radiotherapy	**0**	Pulmonary, peritoneal depositions	KRAS (activating) and PTEN (inactivating)	No	No	Negative (0%)
**Endo-08**	65	Hysterectomy, radiotherapy	**1**	Hepatic, retroperitoneal lymph nodes, peritoneal depositions	No	No	No	Unknown

### Apheresis and vaccinations

For five of the seven patients who underwent apheresis an nDC product could be manufactured. In one patient, the leukapheresis procedure could not be completed because of recurrent interruption in blood flow due to apparent hypercoagulability in the patient. In another patient, although apheresis was successful, the product could not be used because of neutrophilia, associated with an - until that moment - asymptomatic diverticulitis. For two patients, a second apheresis was necessary to obtain enough nDC for the second cycle of vaccination.

Characteristics of the manufactured nDC products are summarized in **Supplementary Figure S2**. In short, products met all prespecified requirements for viability after freezing-thawing, DC purity, and phenotypic maturity. Potency index of batches of nDC product are shown in **Supplementary Table S3**.

Five patients received natural dendritic cell (nDC) vaccinations between February 2018 and February 2021. All five patients were able to complete the pre-planned treatment schedule, including all six vaccinations.

### Adverse events

In general, nDC-vaccinations and chemotherapy were well tolerated. Adverse events are reported in **Table 2**. No serious adverse events (SAE) were observed in any of the patients during the treatment phase. One SAE occurred before treatment started: patient ENDO-07 had intrathoracic bleeding with hemodynamic instability after a tumor biopsy (study procedure-related).

**Table 2:** Adverse events.

AE type	Any grade	Grade 3	Grade 4	Relationship to study procedures or therapy
Alopecia	7	–	–	Related to chemotherapy
Anemia	5	1	0	Related to chemotherapy
Neutrophil count decreased	5	4	1	Related to chemotherapy
Dizziness	4	0	–	Related to chemotherapy
Peripheral sensory neuropathy	4	0	0	Related to chemotherapy
Flu like symptoms	4	0	–	Related to DC vaccination
Fatigue	4	0	0	Related to chemotherapy
Hypokalemia	3	0	0	Related to chemotherapy
Diarrhea	2	0	0	Related to chemotherapy
Nausea	2	0	–	Related to chemotherapy
Tumor pain	1	0	–	Not related to study treatment or procedure
Edema limbs	1	0	–	Related to chemotherapy
Back pain	1	0	–	Not related to study treatment or procedure
Urinary tract infection	1	0	0	Related to chemotherapy
Diverticulitis	1	0	0	Not related to study treatment or procedure
Intrathoracic bleeding	1	1^*^	0	Related to study procedure
Anorexia	1	0	0	Related to chemotherapy
Hidradenitis	1	0	–	Related to chemotherapy

^*^ Serious Adverse Event

High-grade hematological adverse events (CTCAE grade 3 or higher) related to chemotherapy were common. Low-grade non-hematological adverse events (CTCAE grade 1-2) were observed in all patients. The most frequently reported complaints were alopecia, peripheral sensory neuropathy, flu-like symptoms, fatigue and dizziness. Most of these can be attributed to chemotherapy, whereas flu-like symptoms are a well-known side effect of DC vaccination. Four out of five treated patients reported flu-like symptoms, occurring shortly after DC administration and lasting no longer than 48 hours. DC vaccination added little to the toxicity profile of the study treatment.

### Responses to KLH

Before the first vaccination, all five patients who were evaluated for cellular responses to KLH had a proliferation index of < 2, indicating that all patients were naïve to this antigen. After vaccinations with KLH-loaded nDC, four out of five patients had an index of >2. For the group, KLH-response was not significantly increased compared to baseline (p=0.0625). Two of the five patients (ENDO-02 and ENDO-03) had an over four-fold increase, suggesting *de novo* immune responses against this control antigen (**Supplementary Figure S3**).

### Antigen-specific responses

**Figure 2:**
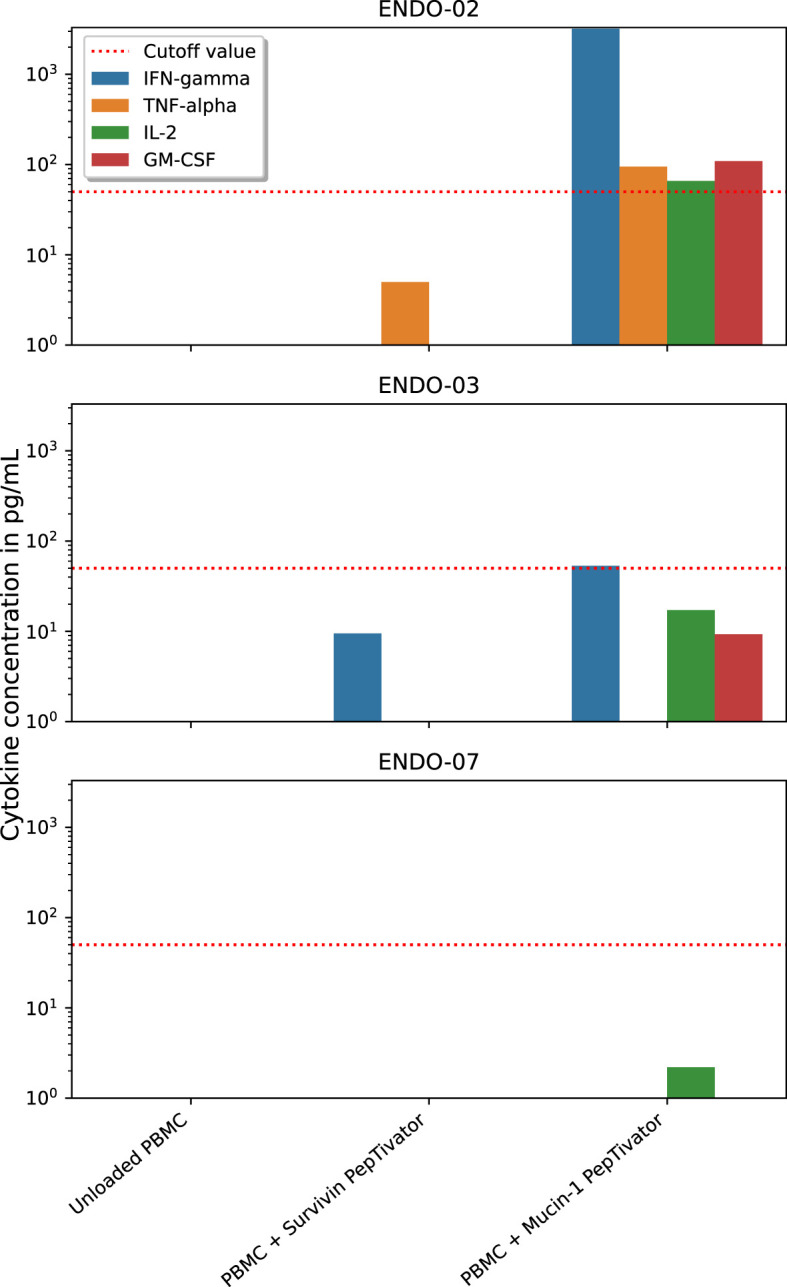
Functionality of antigen-specific T-cells after DC vaccination. dendritic cells (DC), peripheral blood mononuclear cells (PBMC). Skin-infiltrating lymphocytes (SKILs) were cultured from delayed-type hypersensitivity-challenged skin biopsies. Cytokine response of these SKILs in response to coculture with either Mucin-1 PepTivator-pulsed, Survivin PepTivator-pulsed or unpulsed (negative control) autologous PBMCs was measured to assess functional antigen-specific responses.

Sufficient SKILs for analysis of antigen-specificity could be cultured from the skin biopsies of DTH-challenged sites in three of the five patients (ENDO-02, -03 and -07). Only one of these patients matched the HLA-type of the available dextramers, therefore this assay was not performed. SKILs from two of the three patients produced IFN-gamma when cocultured with Mucin-1 PepTivator-pulsed autologous PBMCs (**Figure 2**), but not when cocultured with PBMCs pulsed with Survivin PepTivator or any of the control peptides, suggesting the presence of Mucin-specificity of SKILs. IFN-gamma response was strongest in patient ENDO-02, the patient who also had the strongest KLH-response. Moreover, T-cells from this patient produced TNF-alpha, IL-2 and GM-CSF in response to Mucin-1-pulsed PBMCs.

Dextramers were available for the HLA-types of three patients and for these three patients the presence of antigen-specific CD8 T-cells was assessed in blood samples taken at baseline and after the first cycle of vaccinations. In none of the patients, a clear population of antigen-specific CD8 T-cells could be identified for any of the timepoints.

### Clinical responses

**Figure 3:**
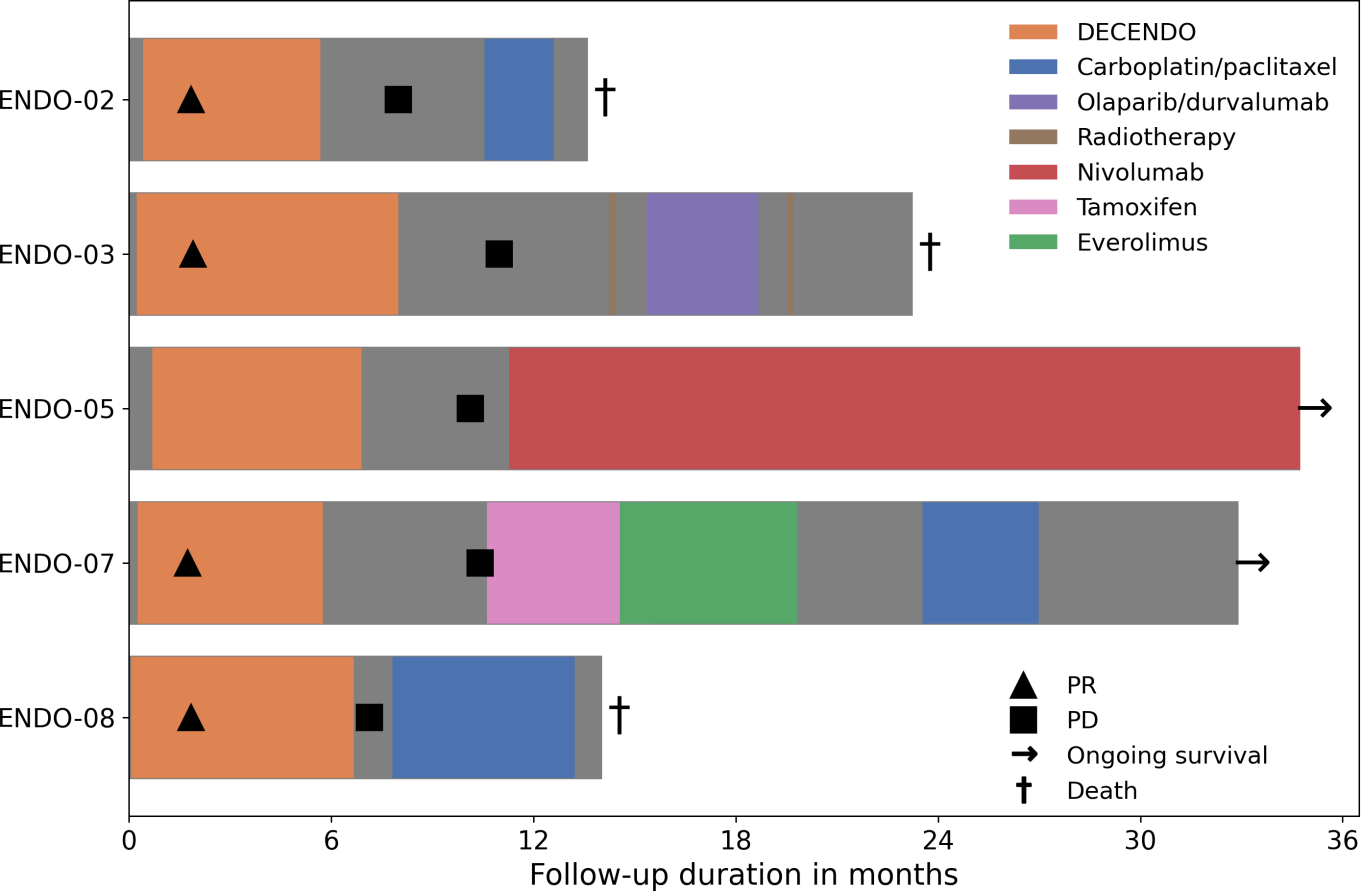
Clinical follow-up during and after the study. PR, partial response; PD, progressive disease.

Clinical response and long-term follow-up data are available for the five patients who were treated with nDC in the study (**Figure 3**). In four of these five patients, a partial response (PR) was observed; one patient had SD. These best responses were all reached after the initial two cycles of chemotherapy.

Median progression free survival (PFS) was 10 months (range: 7 – 11 months). Median overall survival (OS) was 23 months (range: 14 – alive after 35 months). At the date of data cutoff (1-12-2022), two patients were alive: ENDO-05 and ENDO-07. The former was eligible for treatment with ICI because her tumor was determined to be microsatellite instable and she responded well to nivolumab with an ongoing PR; the latter was sequentially treated with tamoxifen, everolimus and carboplatin/paclitaxel chemotherapy.

### Circulating immune cell subsets

Different stimulatory and inhibitory immune cell subsets were measured in peripheral blood from three different timepoints during the study. The first timepoint was at the start of the study, before the start of leukapheresis (week 0 or baseline). The second timepoint was on the day of the 3rd vaccination, 14 days after the last cycle of chemotherapy (week 14 or after DC+CTx). The third timepoint was on the day of the 6th vaccination, roughly 11 weeks after the last chemotherapy (week 23 or after DC).

**Figure 4:**
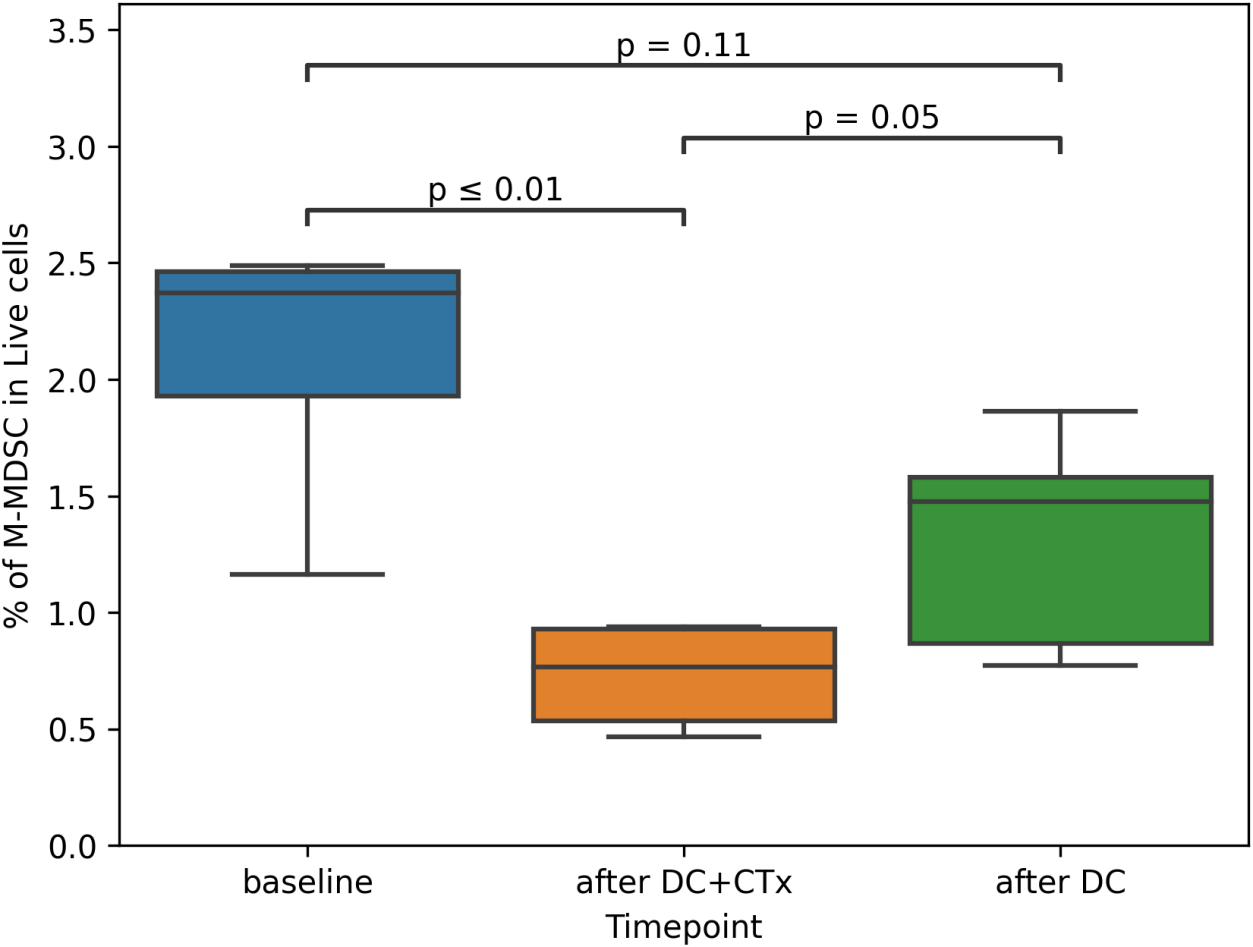
Proportion of M-MDSCs in peripheral blood over time. M-MDSC monocytic myeloid derived suppressor cells, DC dendritic cells, CTx chemotherapy. Percentage of M-MDSCs was assessed in peripheral blood mononuclear cells obtained from patients at baseline, on the day of the third vaccination (after DC+CTx) and on the day of the sixth vaccination (after DC). Paired t-tests were used to compare differences between timepoints.

Of most interest was whether the frequency of MDSCs and Tregs changed in response to treatment. At baseline, median frequency of M-MDSCs was 2.4% of PBMCs (range: 1.2 - 2.5%) and the median frequency of Tregs was 2.4% of CD4 T-cells (range: 1.4 - 3.5%). After DC+CTx, monocytic MDSC (M-MDSC) counts were significantly decreased to 0.8% (range: 0.5 - 0.9%) (**Figure 4**). No differences were observed between timepoints for the other immunosuppressive subsets, such as Tregs and early MDSCs (eMDSC), as well as immune effector cells (HLA-DR+ monocytes, CD8 and CD4 T-cells, (**Supplementary Figure S4**). The frequencies of different conventional DC subsets cDC1, cDC2 and cDC3 as well as pDCs remained generally stable over time, cDC1 being exceedingly rare (median 0.04%, range: 0.00 - 0.06%) compared to the other DC subsets. Overall, circulating DCs had an immature phenotype at baseline except for cDC3, of which 42% were CD83+ (median, range: 24 - 52%). For cDC1, cDC2 and pDC, this was 3%, 11.6%, and 1.4%, respectively.

Finally, expression of co-stimulatory (OX40, ICOS) and co-inhibitory (PD-1, PD-L1, TIM-3, LAG-3, TIGIT) immune checkpoints on CD4 and CD8 T-cells was measured. In short, no trend for change in percentage of cells positive for any of the checkpoints could be observed that was consistent between patients. Analysis of checkpoint expression across patients and timepoints shows that PD-1 was most abundantly expressed on both CD8 and CD4 T-cells, followed by TIGIT, which was more expressed on CD8 T-cells. The immune stimulatory checkpoint OX40 was expressed on 25% of CD4 T-cells compared to only 4% of CD8 T-cells. Mean expression of the other immune checkpoints (PD-L1, TIM-3, ICOS and LAG-3) were all below 5%.

To further explore interindividual differences that could help explain differences in clinical and immunological responses to therapy, a heatmap was drawn for various characteristics across patients (**Supplementary Figure S5** shows the relative abundance of immune cell subsets, and levels of marker expression on selected immune cells). Patient ENDO-02, who showed the best immunological response had a relatively high percentage of total T-cells and CD4 T-cells, but not CD8 T-cells. Patient ENDO-5, the only participant with not at least a PR, but who responded well to subsequent treatment with anti-PD-1 therapy, had a remarkable immune checkpoint expression profile: expression of costimulatory checkpoints ICOS and OX-40 as well as PD-L1, TIM-3, LAG-3 were higher compared to other patients. Interestingly, PD-1 expression was close to average.

### Multiplex immunohistochemistry

**Figure 5:**
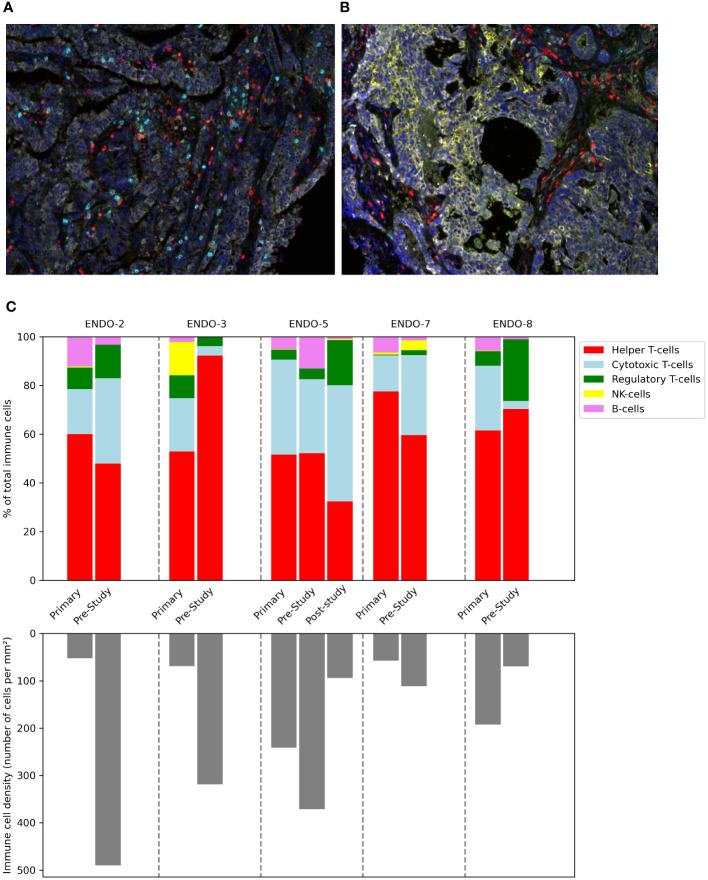
Composition of the immune infiltrate of tumor before, during and after the study. Tumor samples taken during hysterectomy (for primary tumors) or image-guided biopsy (for pre-study and post-study (after progression on study treatment) samples) were stained and analyzed using multiplex immunohistochemistry. Multispectral images at 20× magnification stained with a multiplex panel containing CD3, CD8, FoxP3, CD20, CD56, DAPI and cytokeratin of a hysterectomy sample **(A)** and a tumor biopsy **(B)**. **(C)** Immune cell density in tumor samples.

In addition to the tumor biopsies that were taken at baseline for this study, we also assessed archived material from primary tumors (available for all patients) and biopsies that were taken post-study for other reasons (available for one patient). The composition of the immune infiltrate is shown in **Figure 5**. Substantial differences were present between patients and within patients across the different samples. Notably, the patient with the shortest PFS (ENDO-08) had by far the highest proportion of Tregs, followed by the patient with the second shortest PFS. In the only available post-study sample (for ENDO-05, obtained after progression on study treatment and before initiation of subsequent treatment with nivolumab), an increase in the proportion of Tregs as well as CD8 T-cells was seen compared to pre-study.

## Discussion

In this exploratory phase I/II study, we investigated the immunological efficacy of nDC vaccination combined with platinum-based chemotherapy in endometrial cancer patients, as well as the safety and feasibility of this approach.

As in previous trials with (n)DC-vaccination, the safety profile was excellent. No DC vaccination-related AEs of CTCAE grade >2 were observed. Specifically, there were no signs of autoimmunity. In addition, the combinatory nDC vaccination/chemotherapy approach proved feasible. Patient-related factors hampered successful leukapheresis or production of an nDC product in two out of seven patients (29%), a higher percentage than in previous trials (compare 2% in the MIND-DC-trial, unpublished data). This most likely reflects the higher morbidity of these patients who, despite having a WHO performance status of 0 or 1, have an advanced malignancy and all received prior treatment. The experience from this small cohort should be extrapolated only with caution, but it is clear that patient selection for this treatment approach is important. Administration of the combined treatment itself proved to be feasible: all patients who received a first vaccination could complete all six planned vaccinations.

In antigen-specific immunotherapy, appropriate antigen selection is crucial. In this study, Mucin-1 and Survivin were chosen based on their alignment with endometrial tumor antigens. All screened patients expressed both these antigens in their tumor tissue, making the combination a promising choice to induce a broad anti-tumoral immune response. The other main issue is immunogenicity. In our study, an antigen-specific response could be demonstrated in only two out of five patients. Interestingly, demonstrable antigen-specific cells coincided with the strongest responses against the control antigen KLH. In contrast, in a recent trial we conducted with a similar nDC product in stage III melanoma patients, all participants mounted a response against the control antigen (16). A possible explanation is that tumor-mediated suppression of the immune system, either systemic or in the injected inguinal lymph node, in these mEC patients interfered with effective T-cell priming.

In contrast with earlier trials, in which we observed a correlation between immunological responses and clinical benefit (17, 37), the two immunological responders did not have a more favorable clinical outcome than non-responders. A possible reason for the absence of clinical response given an immunological response is inherent or acquired low cytotoxic activity of the induced T-cells. We did not directly evaluate the inherent cytotoxic activity of cultured T-cells, but tested functionality by means of measuring cytokine responses. Functional impairment of CD8 T-cells could be induced by cancer-associated humoral or cellular factors, some of which (like MDSCs and Tregs) were identified during the study. Another explanation could be swift downregulation of the target antigen on tumor cells, which we did not account for.

The multiplex IHC data give additional insight into the state of the immune system intratumorally. However, because for most patients, no tumor tissue was available for analysis after DC vaccination combined with chemotherapy, any findings could at best be predictive of a response. In line with what is known from the literature, a low proportion of CD8 T-cells as well as a high number of Tregs coincide with poor prognosis in this small cohort. Interestingly, in the only post-study biopsy available (for patient ENDO-5), we observed an increase in intratumoral CD8 T-cells as well as an increase in percentage of Tregs in response to study treatment. This patient later experienced a long-lasting response to anti-PD1 blocking therapy, which can counteract PD-L1-mediated Treg activity (38). In this patient, initial priming of T-cells by the DC vaccine might have enhanced the effectiveness of the subsequent immune checkpoint blockade. The combination of these treatment modalities could be beneficial in patients where 1) priming naive T-cells by DC vaccination alone is not enough to overcome cancer-induced immunosuppression and 2) the existing repertoire of primed T-cells is not sufficient for checkpoint inhibition alone to be effective. This could improve response rates for checkpoint blockade in mismatch repair proficient (pMMR) mEC and should be explored in future trials.

We planned nDC-vaccinations on day 14 after chemotherapy, based on previous studies showing that the balance between suppressive and effector immune cells around that time is optimal to induce strong vaccine responses (39). Indeed, the data from the present study are in line with previous findings that cisplatin selectively depletes M-MDSC counts after two weeks, at which time the numbers of pro-inflammatory and effector immune cells have recovered (28). We now show that the same dynamic occurs in response to carboplatin/paclitaxel. For now, when given in combination, 14 days after platinum-based chemotherapy seems to be the best moment for administering any therapeutic cancer vaccine.

The finding that circulating numbers of DC subsets, and in particular cDC1, generally remain stable during therapy, is important with regard to future DC + CTx combination studies. As mentioned before, the rare cDC1s are in theory the ideal cellular subset for DC based immunotherapy. Their capacity for cross-presenting exogenous material via MHC class I to CD8 T-cells make them well-suited for use with tumor lysate as a (neo)antigen source. Our data suggest that a fair yield of cDC1 can be expected of repeated leukapheresis even after chemotherapy if the result from one apheresis does not suffice. This paves the road for future combination studies with cDC1 and chemotherapy.

In conclusion, the present study shows that therapeutic DC vaccination can be safely combined with conventional chemotherapy for patients with metastatic endometrial cancer. It also shows that this approach can induce specific immune responses against tumor antigens in some patients. The translational findings support the rationale for combinatory treatment with DC vaccination and chemotherapy. Future studies should investigate how to optimize the efficacy of DC vaccination in combination with chemotherapy in endometrial cancer patients.

## Data availability statement

The raw data supporting the conclusions of this article will be made available by the authors, without undue reservation.

## Ethics statement

The studies involving humans were approved by Dutch Central Committee on Research Involving Human Subjects. The studies were conducted in accordance with the local legislation and institutional requirements. The participants provided their written informed consent to participate in this study.

## Author contributions

BK: Writing – original draft, Writing – review & editing, Data curation, Investigation, Visualization. GS: Conceptualization, Investigation, Methodology, Supervision, Writing – review & editing. MG: Methodology, Software, Writing – review & editing. SH-B: Investigation, Writing – review & editing. HW: Writing – review & editing. PO: Conceptualization, Funding acquisition, Investigation, Supervision, Writing – review & editing. ID: Conceptualization, Funding acquisition, Investigation, Supervision, Writing – review & editing.

## Supplementary material

Supplementary Table 1 Antibodies used in flow cytometry.

Supplementary Table 2 Antibodies/Opals used in multiplex immunohistochemistry staining.

Supplementary Table 3 Potency index of DC product.

Supplementary Figure 1 Antigen expression on tumor tissue. Tumor biopsies were stained with antibodies against the vaccine antigens Survivin and Mucin-1. Examples of immunohistochemical staining images are shown for positive control tissue and for patient ENDO-04 and ENDO-07.

Supplementary Figure 2 Phenotype and purity of the DC product. DC dendritic cell. **(A)** yield of mature, antigen-loaded nDCs, **(B)** viability, **(C)**, purity, based on percentage of cells expressing the combination of CD123 and BDCA2 (plasmacytoid DC) or CD1c in the absence of CD20 (conventional DC), **(D)** marker expression on non-DCs, **(E)** phenotype of DCs.

Supplementary Figure 3 KLH-specific responses against the control antigen KLH before and after vaccination. KLH Keyhole Limpet Hemocyanin. Peripheral blood mononuclear cells (PBMCs) were analyzed for the presence of KLH-specific T-cells using a proliferation assay before the first vaccination and after DC vaccination. Proliferation index was defined as proliferation with KLH/proliferation without KLH. The maximal index during DC vaccination therapy is shown for each patient (ENDO-02: day of vaccination 6, ENDO-03: day of first DTH, ENDO-05: day of vaccination 4, ENDO-07: day of vaccination 6, ENDO-08: day of vaccination 2). A Wilcoxon signed-rank test was used to compare responses before and after vaccination.

Supplementary Figure 4 Longitudinal changes in circulating immune cell subsets. Peripheral blood mononuclear cells (PBMCs) were obtained from patients at baseline, on the day of the third vaccination (after DC+CTx) and on the day of the sixth vaccination (after DC) and marker expression plotted for each patient. Using flow cytometry, percentages of different immune cell subsets and levels of marker expression on cells were assessed. Graphs show longitudinal changes for each patient.

Supplementary Figure 5 Interindividual differences between patients in terms of the percentage of immune cell subsets in peripheral blood **(A)** and expression of relevant markers on immune cells **(B)** prior to study treatment. Data were normalized per characteristic (frequency of immune cell subset or percentage positive for marker) before a heatmap was plotted. Legend bar numbers indicate standard deviations above or below the average for each characteristic.

Supplementary Figure 6 Best overall response: Change in tumor diameter (percentage) according to RECIST v 1.1.
