# Supplementary material for: Dendritic cell vaccination combined with carboplatin/paclitaxel for metastatic endometrial cancer patients: results of a phase I/II trial
Source: Front Immunol. 2024 Feb 20;15:1368103. doi: 10.3389/fimmu.2024.1368103 (PMC10912556; doi:10.3389/fimmu.2024.1368103)
Supplement: Supplementary Table 1 — Antibodies used in flow cytometry. [file Table_1.docx]

**Table S1 -** Antibodies used in flow cytometry

| **Panel** | **Target** | **Fluorophore** | **Antibody clone** | **Manufacturer** |
| --- | --- | --- | --- | --- |
| **Vaccine purity and phenotype panels** | CD1c | Viobright FITC | AD5-8E7 | Miltenyi Biotec |
|  | BDCA2 | PE | AC144 | Miltenyi Biotec |
|  | CD123 | APC | AC145 | Miltenyi Biotec |
|  | CD20 | PE-Vio770 | LT20 | Miltenyi Biotec |
|  | CD45 | APC-Vio770 | 5B1 | Miltenyi Biotec |
|  | CD14 | Viogreen | Tuk4 | Miltenyi Biotec |
|  | FcεRI | VioBlue | CRA-1 | Miltenyi Biotec |
|  | CD14 | FITC | Tuk4 | Miltenyi Biotec |
|  | CD15 | PE | VIMC6 | Miltenyi Biotec |
|  | CD56 | APC | AF12-7H3 | Miltenyi Biotec |
|  | CD3 | VioBlue | BW264/56 | Miltenyi Biotec |
|  | HLA-ABC | APC | REA230 | Miltenyi Biotec |
|  | HLA-DR/DP/DQ | APC | REA332 | Miltenyi Biotec |
|  | CCR7 | APC | REA108 | Miltenyi Biotec |
|  | CD80 | APC | 2D10 | Miltenyi Biotec |
|  | CD83 | APC | HB15 | Miltenyi Biotec |
|  | CD86 | APC | FM95 | Miltenyi Biotec |
| **Checkpoint panel** | CD8 | FITC | BW135/80 | Miltenyi Biotec |
|  | TIM-3 | PE | 7D3 | BD Biosciences |
|  | ICOS | PerCP-Cy5.5 | DX29 | BD Biosciences |
|  | CD3 | PE-Cy7 | UCHT1 | eBioscience |
|  | OX40 | APC | ACT35 | Invitrogen |
|  | CD4 | APC R700 | RPA-T4 | BD Biosciences |
|  | PD-1 | BV421 | EH12.2H7 | Biolegend |
|  | LAG3 | superbright 600 | 3DS223H | Invitrogen |
|  | PD-L1 | BV711 | 29E.2A3 | Biolegend |
|  | TIGIT | BV786 | A15153G | Biolegend |
| **DC panel** | Clec9a | VioBright FITC | 8F9 | Miltenyi |
|  | BDCA-2 | PE | AC144 | Miltenyi Biotec |
|  | CD14 | PerCP | Tük4 | Miltenyi |
|  | HLA-DR | PE-Cy7 | L243 | BD Biosciences |
|  | BDCA-3 | APC | REA674 | Miltenyi |
|  | CD123 | Alexa Fluor 700 | 6H6 | Biolegend |
|  | CD163 | BV421 | GHI/61 | BD Biosciences |
|  | CD45 | VioGreen | 5B1 | Miltenyi Biotec |
|  | CD20 | BV711 | 2H7 | Biolegend |
|  | CD83 | BV785 | HB15e | Biolegend |
| **MDSC panel** | Lineage:  CD3  CD19  CD20  CD56 | FITC | SK7  SJ25C1  L27  NCAM 16.2 | BD Biosciences  BioLegend  BD Biosciences  BD Biosciences |
|  | CD33 | PE | MOPC-21 | BioLegend |
|  | PD-L1 | PE-Cy7 | MIH1 | BD Biosciences |
|  | CD123 | Alexa Fluor 700 | 6H6 | BioLegend |
|  | HLA-DR | BV510 | L243 | BioLegend |
|  | CD11b | BV605 | ICRF44 | BD Biosciences |
|  | CD14 | BV786 | M5E2 | BD Biosciences |
|  | Arg1 | APC | A1exF5 | Invitrogen |
|  | IDO | BV421 | eyedio | Invitrogen |
| **Treg panel** | CTLA-4/CD152 | PE | BNI3 | BD Biosciences |
|  | CD8 | PerCP-Cy5.5 | SK1 | BD Biosciences |
|  | CD45RO | PE-Cy7 | UCHL1 | BD Biosciences |
|  | CCR7 | APC | REA108 | Miltenyi Biotec |
|  | CD25 | BV421 | M-A251 | BD Biosciences |
|  | CD4 | BV510 | RPA-T4 | BioLegend |
|  | CD3 | BV605 | UCHT1 | BioLegend |
|  | FoxP3 | FITC | PCH101 | eBioscience |
