# Supplementary material for: Dendritic cell vaccination combined with carboplatin/paclitaxel for metastatic endometrial cancer patients: results of a phase I/II trial
Source: Front Immunol. 2024 Feb 20;15:1368103. doi: 10.3389/fimmu.2024.1368103 (PMC10912556; doi:10.3389/fimmu.2024.1368103)
Supplement: Supplementary Table 2 — Antibodies/Opals used in multiplex immunohistochemistry staining. [file Table_2.docx]

**Table S2 -** Antibodies / Opals used in multiplex immunohistochemistry staining

| **Antibody** | **Clone** | **Dilution** | **Opal** |
| --- | --- | --- | --- |
| anti-CD56 | MRQ-42 | 1/1500 | Opal620 |
| anti-CD8 | C8/144B | 1/200 | Opal690 |
| anti-CD20 | L26 | 1/300 | Opal480 |
| anti-CD3 | Sp7 | 1/200 | Opal520 |
| anti-Foxp3 | 236A/E7 | 1/100 | Opal570 |
| anti-pan-cytokeratin |  |  | Opal780 |
