# Supplementary material for: Dendritic cell vaccination combined with carboplatin/paclitaxel for metastatic endometrial cancer patients: results of a phase I/II trial
Source: Front Immunol. 2024 Feb 20;15:1368103. doi: 10.3389/fimmu.2024.1368103 (PMC10912556; doi:10.3389/fimmu.2024.1368103)
Supplement: Supplementary Table 3 — Potency index of DC product. [file Table_3.docx]

**Table S3** – Potency index of DC product

| Patient | Batch number | Potency Index |
| --- | --- | --- |
| ENDO-02 | A19D1701 | 19 |
| ENDO-03 | A19F1901 | 17 |
| ENDO-03 | A19M0904 | 10 |
| ENDO-05 | A19M1601 | 8 |
| ENDO-07 | A20C0201 | 4 |
| ENDO-08 | A20G2701 | 10 |
| ENDO-08 | A20M2101 | 7 |
