# Supplementary figures and images for: Dendritic cell vaccination combined with carboplatin/paclitaxel for metastatic endometrial cancer patients: results of a phase I/II trial
Source: Front Immunol. 2024 Feb 20;15:1368103. doi: 10.3389/fimmu.2024.1368103 (PMC10912556; doi:10.3389/fimmu.2024.1368103)

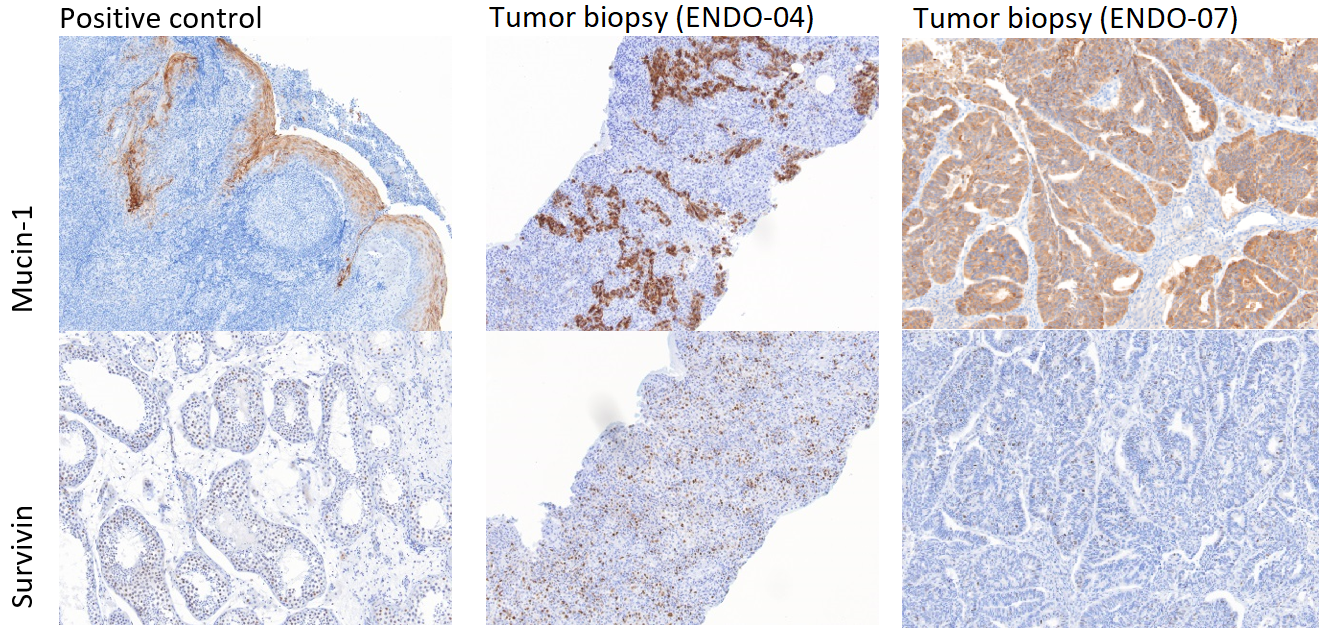

Supplement: Supplementary Figure 1 — Antigen expression on tumor tissue. Tumor biopsies were stained with antibodies against the vaccine antigens Survivin and Mucin-1. Examples of immunohistochemical staining images are shown for positive control tissue and for patient ENDO-04 and ENDO-07. [file Image_1.tiff]

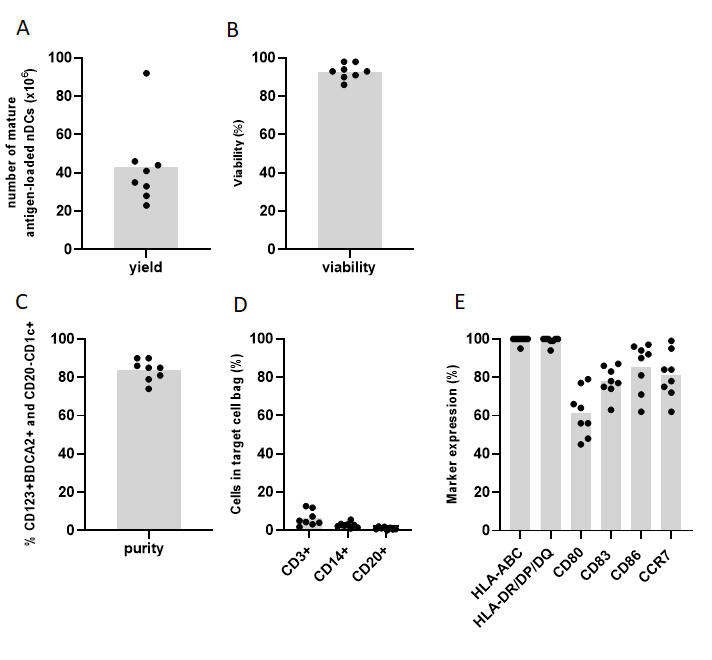

Supplement: Supplementary Figure 2 — Phenotype and purity of the DC product. DC dendritic cell. (A) yield of mature, antigen-loaded nDCs, (B) viability, (C), purity, based on percentage of cells expressing the combination of CD123 and BDCA2 (plasmacytoid DC) or CD1c in the absence of CD20 (conventional DC), (D) marker expression on non-DCs, (E) phenotype of DCs. [file Image_2.tiff]

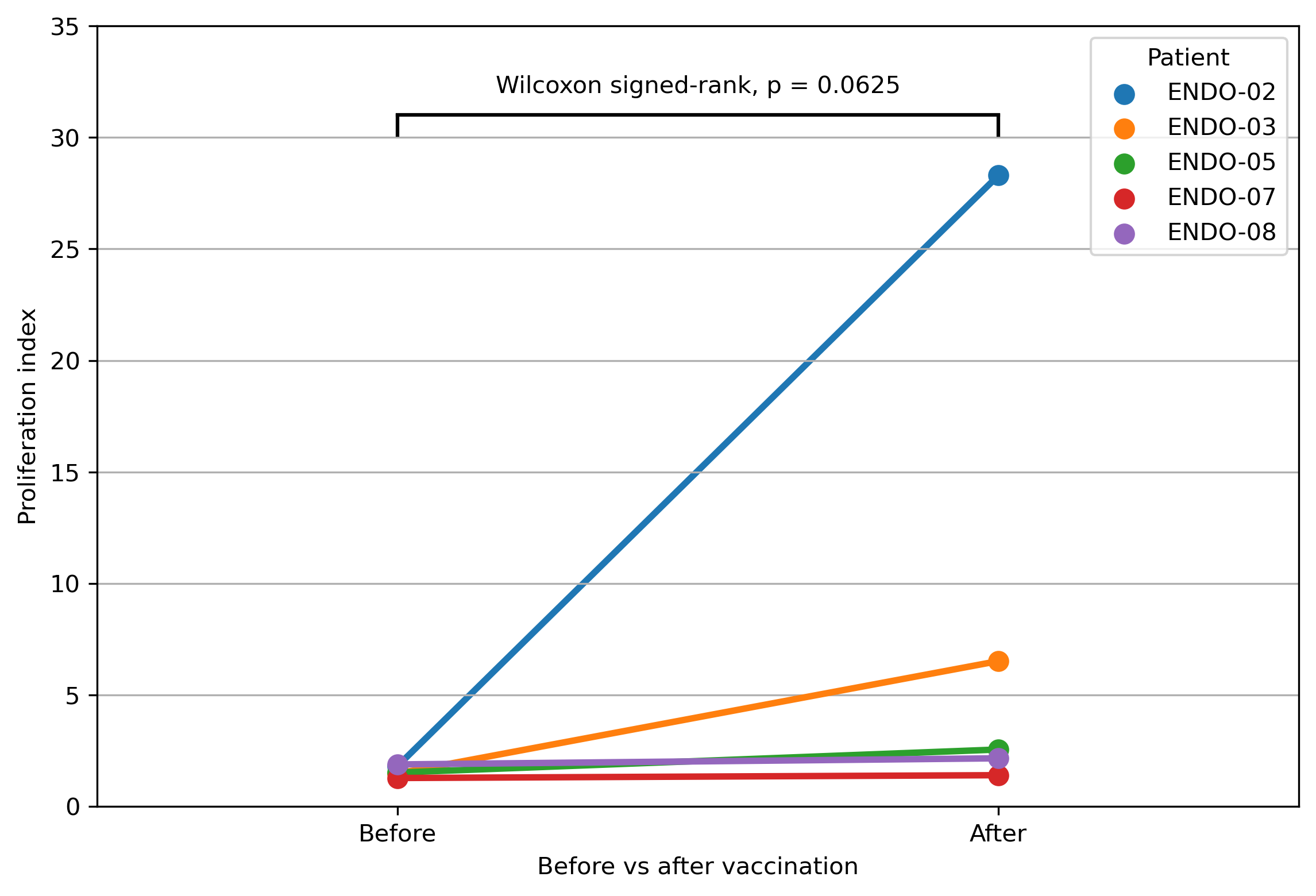

Supplement: Supplementary Figure 3 — KLH-specific responses against the control antigen KLH before and after vaccination. KLH Keyhole Limpet Hemocyanin. Peripheral blood mononuclear cells (PBMCs) were analyzed for the presence of KLH-specific T-cells using a proliferation assay before the first vaccination and after DC vaccination. Proliferation index was defined as proliferation with KLH/proliferation without KLH. The maximal index during DC vaccination therapy is shown for each patient (ENDO-02: day of vaccination 6, ENDO-03: day of first DTH, ENDO-05: day of vaccination 4, ENDO-07: day of vaccination 6, ENDO-08: day of vaccination 2). A Wilcoxon signed-rank test was used to compare responses before and after vaccination. [file Image_3.tif]

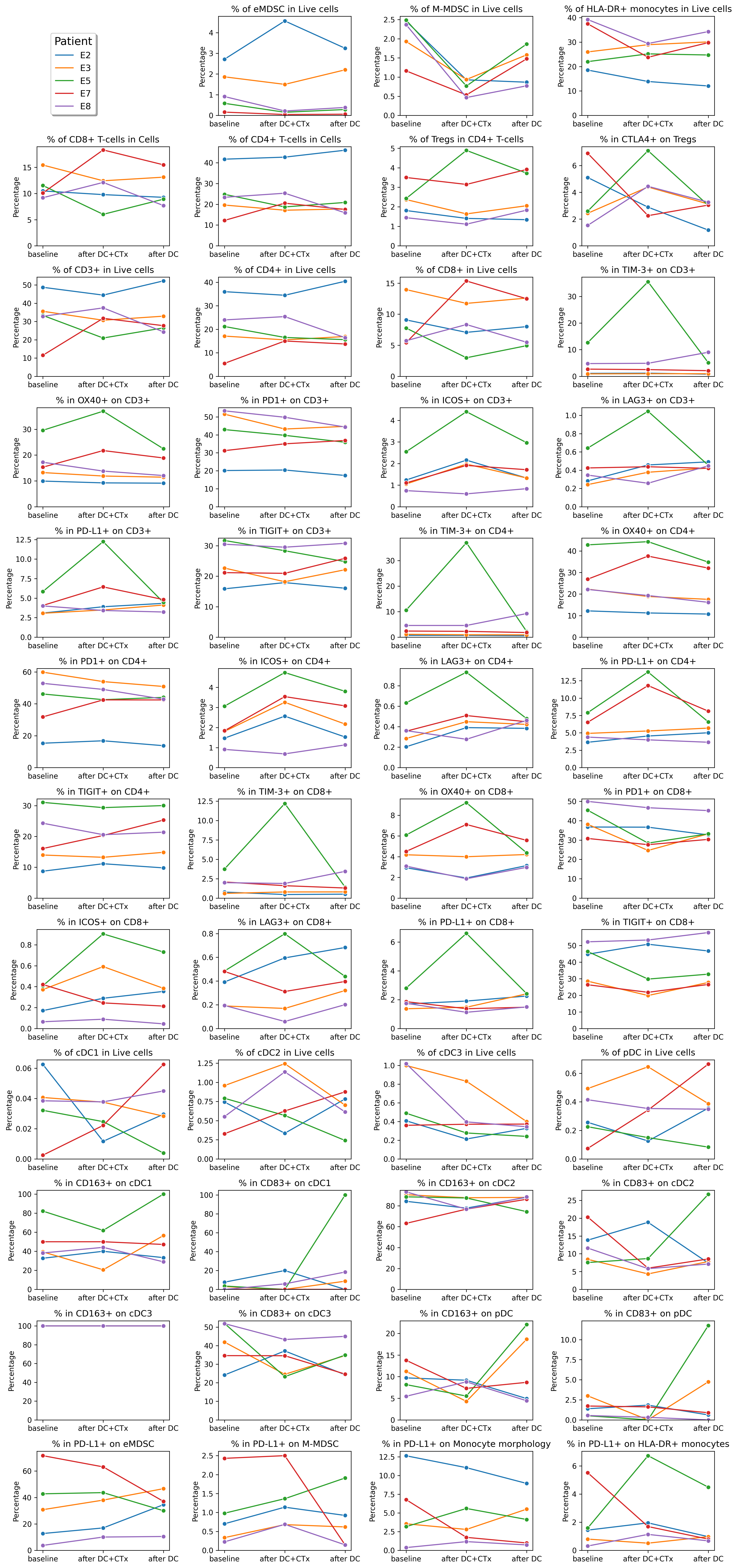

Supplement: Supplementary Figure 4 — Longitudinal changes in circulating immune cell subsets. Peripheral blood mononuclear cells (PBMCs) were obtained from patients at baseline, on the day of the third vaccination (after DC+CTx) and on the day of the sixth vaccination (after DC) and marker expression plotted for each patient. Using flow cytometry, percentages of different immune cell subsets and levels of marker expression on cells were assessed. Graphs show longitudinal changes for each patient. [file Image_4.tiff]

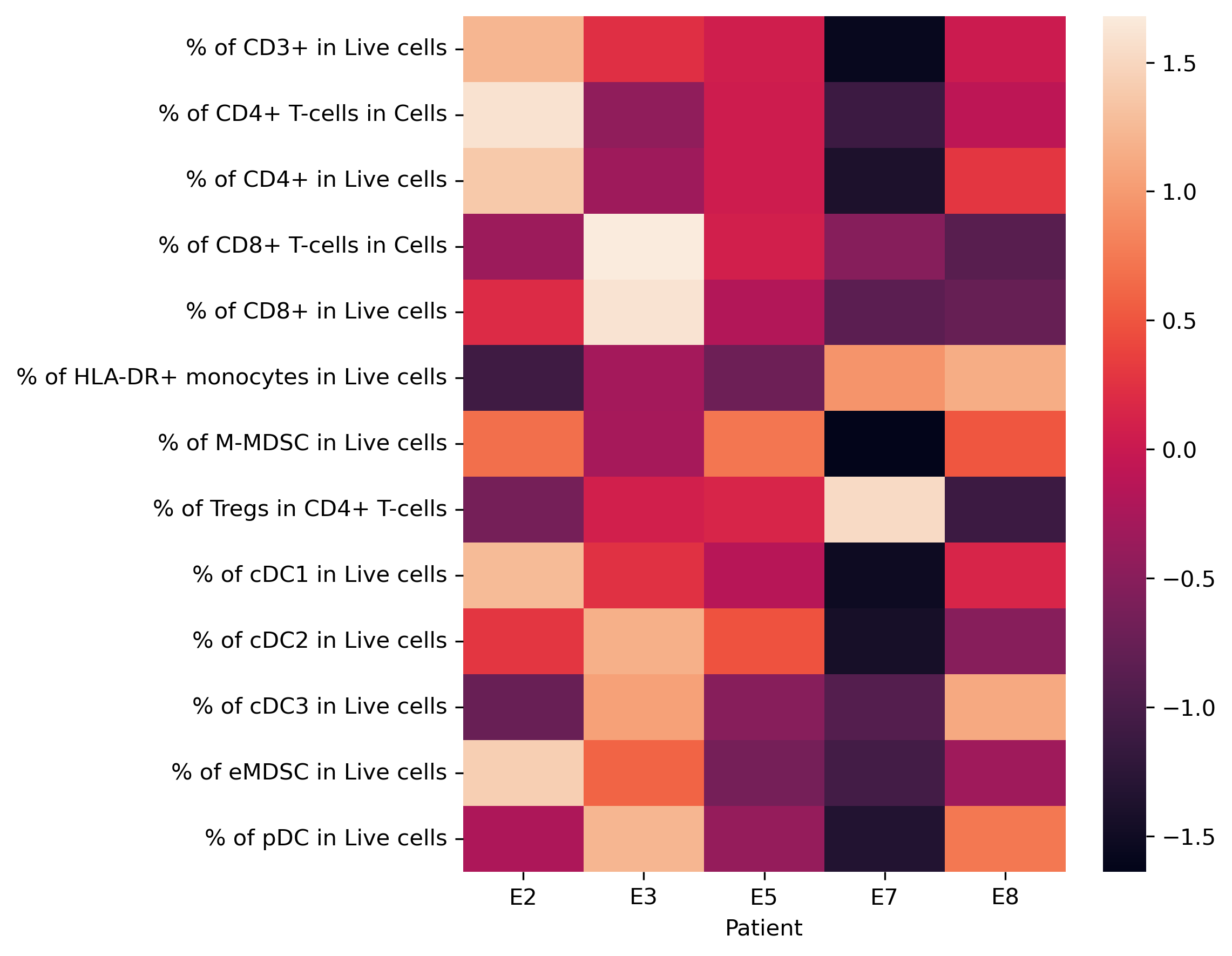

Supplement: Supplementary Figure 5 — Interindividual differences between patients in terms of the percentage of immune cell subsets in peripheral blood (A) and expression of relevant markers on immune cells (B) prior to study treatment. Data were normalized per characteristic (frequency of immune cell subset or percentage positive for marker) before a heatmap was plotted. Legend bar numbers indicate standard deviations above or below the average for each characteristic. [file Image_5.tiff]

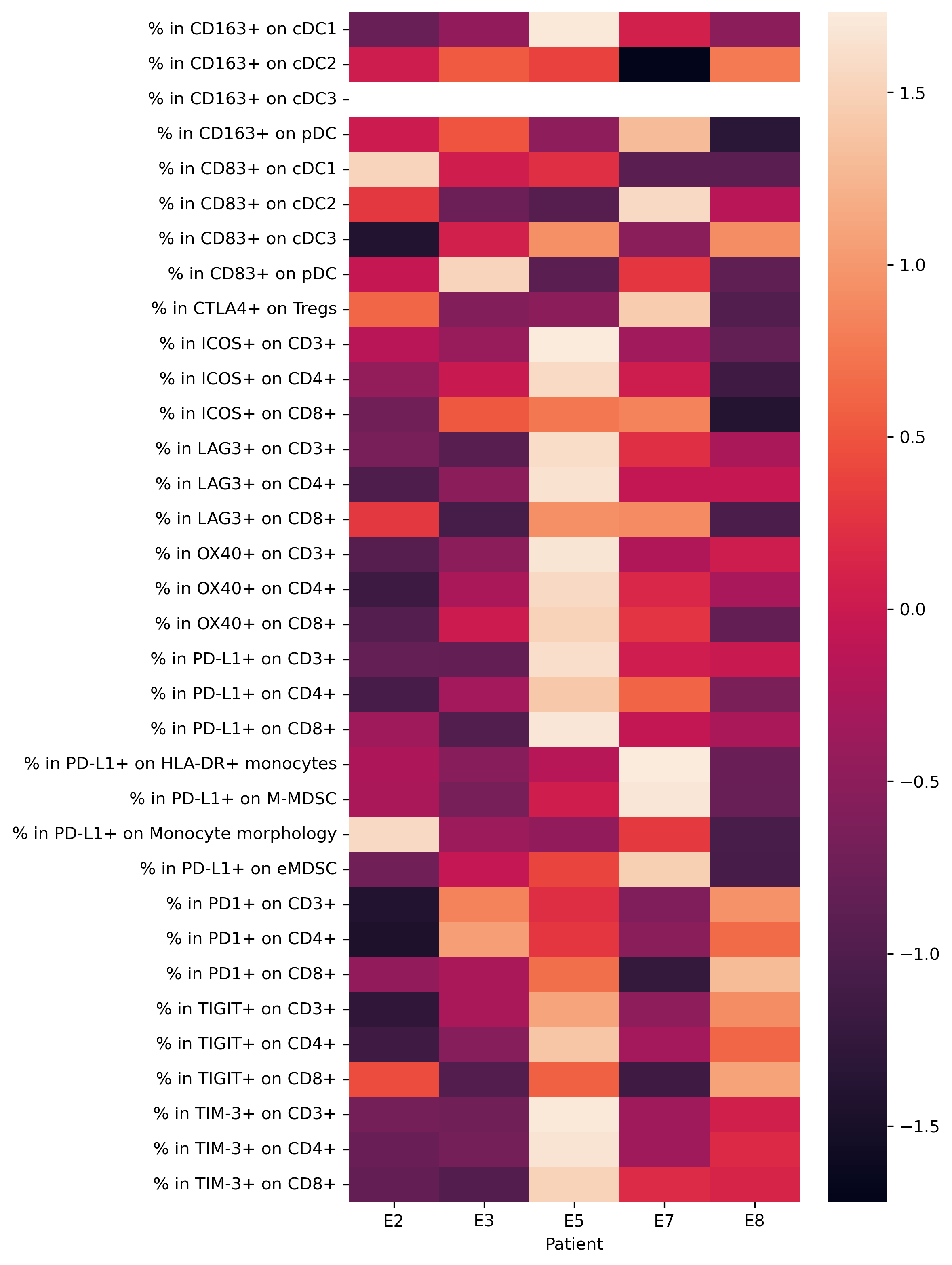

Supplement: Supplementary Figure 6 — Best overall response: Change in tumor diameter (percentage) according to RECIST v 1.1. [file Image_6.tiff]

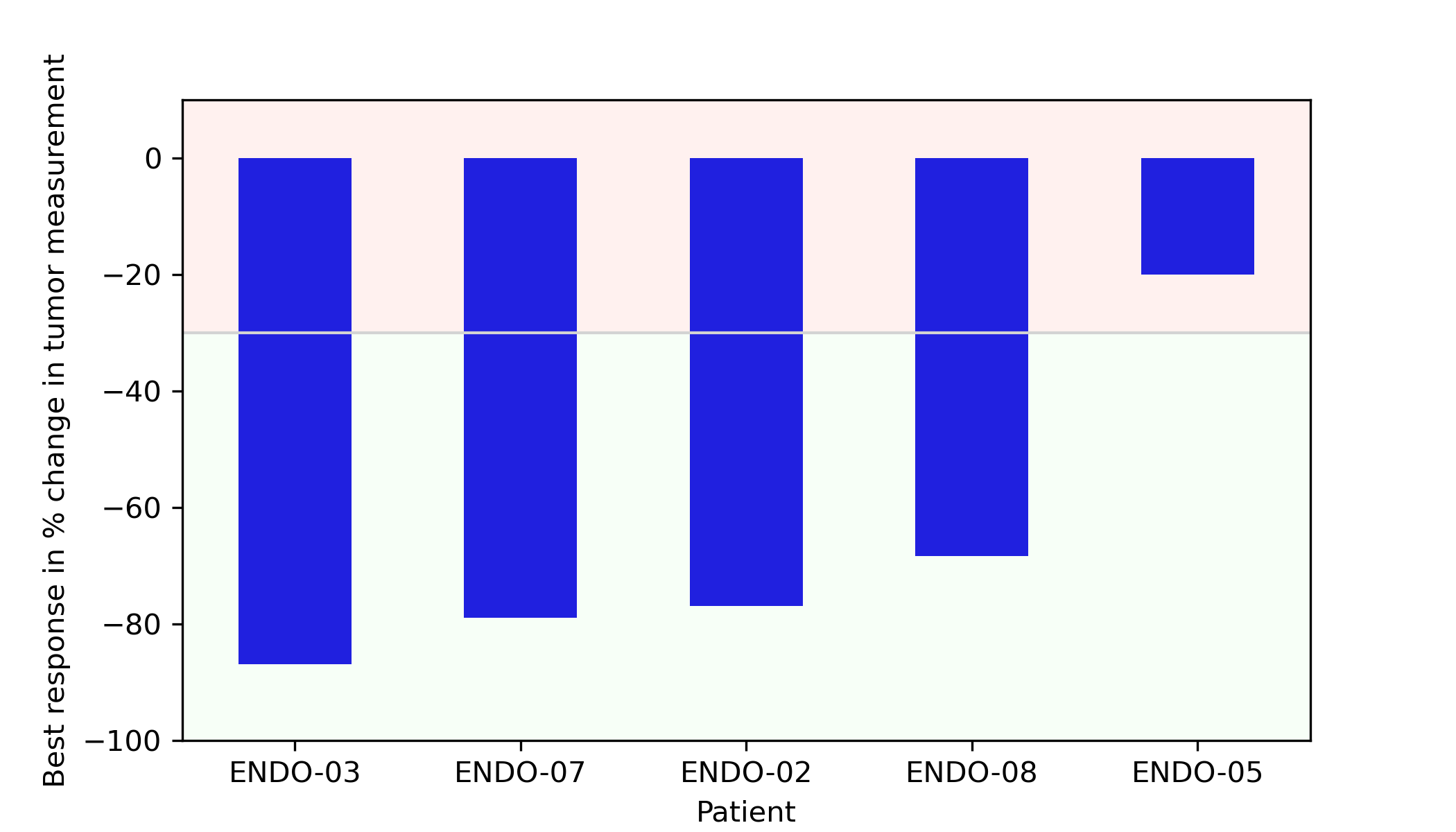

Supplement: Supplementary file 10 [file Image_7.tiff]
